# Eco-Friendly Design of Chitosan-Based Films with Biodegradable Properties as an Alternative to Low-Density Polyethylene Packaging

**DOI:** 10.3390/polym16172471

**Published:** 2024-08-30

**Authors:** Johanna Fiallos-Núñez, Yaniel Cardero, Gustavo Cabrera-Barjas, Claudio M. García-Herrera, Matías Inostroza, Miriam Estevez, Beatriz Liliana España-Sánchez, Loreto M. Valenzuela

**Affiliations:** 1 Departamento de Ingeniería Química y Bioprocesos, Pontificia Universidad Católica de Chile, Santiago 6904411, Chile; jefiallos@uc.cl (J.F.-N.); ycardero@uc.cl (Y.C.); 2 Facultad de Ciencias para el Cuidado de la Salud, Universidad San Sebastián, Lientur 1439 Región del Biobío, Concepción 4080871, Chile; gustavo.cabrera@uss.cl; 3 Departamento de Ingeniería Mecánica, Universidad de Santiago de Chile, Santiago 9170020, Chile; claudio.garcia@usach.cl (C.M.G.-H.); matias.inostroza.i@usach.cl (M.I.); 4 Centro de Física Aplicada y Tecnología Avanzada (CFATA), Universidad Nacional Autónoma de México, Boulevard Juriquilla 3001, Querétaro 76230, Mexico; miries@fata.unam.mx; 5 Centro de Investigación y Desarrollo Tecnológico en Electroquímica (CIDETEQ) S. C., Parque Tecnológico Querétaro s/n, Sanfandila, Pedro Escobedo, Querétaro 76703, Mexico

**Keywords:** eco-friendly packaging, chitosan-based films, mechanical performance, biodegradable packaging, undegradable LDPE

## Abstract

Biopolymer-based films are a promising alternative for the food packaging industry, in which petrochemical-based polymers like low-density polyethylene (LDPE) are commanding attention because of their high pollution levels. In this research, a biopolymer-based film made of chitosan (CS), gelatin (GEL), and glycerol (GLY) was designed. A Response Surface Methodology (RSM) analysis was performed to determine the chitosan, gelatin, and glycerol content that improved the mechanical properties selected as response variables (thickness, tensile strength (TS), and elongation at break (EAB). The content of CS (1.1% *w*/*v*), GEL (1.1% *w*/*v*), and GLY (0.4% *w*/*v*) in the film-forming solution guarantees an optimized film (OPT-F) with a 0.046 ± 0.003 mm thickness, 11.48 ± 1.42 mPa TS, and 2.6 ± 0.3% EAB. The OPT-F was characterized in terms of thermal, optical, and biodegradability properties compared to LDPE films. Thermogravimetric analysis (TGA) revealed that the OPT-F was thermally stable at temperatures below 300 °C, which is relevant to thermal processes in the food industry of packaging. The reduced water solubility (WS) (24.34 ± 2.47%) and the improved biodegradability properties (7.1%) compared with LDPE suggests that the biopolymer-based film obtained has potential applications in the food industry as a novel packaging material and can serve as a basis for the design of bioactive packaging.

## 1. Introduction

Nowadays, most of the materials used in food packaging are petrochemical-based polymers due to their low cost and excellent barrier properties. However, petrochemical-based polymers are non-biodegradable, increasing environmental concerns regarding long-term pollution [1]. LDPE is broadly used as packaging for food products due to its excellent barrier and mechanical properties [2]. Based on this, the development of biomaterials for food packaging has attracted the attention of government regulations [3]. As a consequence, the production of biodegradable films that can be used as packaging material has potentially increased, with biopolymers like carbohydrates, lipids, proteins, or their blends being commonly used [4].

Gelatin-based films are widely used because of their excellent film-forming capability and good barrier properties to CO_2_ and O_2_. However, gelatin’s direct application in food matrices is limited due to its swelling properties and its dissolution upon contact with food. To avoid these problems, blending gelatin with lipids or another film-forming material [5] or with an opposite-charge polymer improves the physical and mechanical properties of the gelatin-based films, resulting in a film with a higher performance [3]. Chitosan is a polycationic polymer obtained from the deacetylation of chitin, the second-most prominent polysaccharide in nature [6]. Chitosan has attractive characteristics like film-forming ability, biodegradability, and antioxidant and antimicrobial activity, and it is an excellent barrier to the permeation of O_2_. It is broadly used in food preservation against spoilage deterioration [7].

On the other hand, the degree of deacetylation of chitosan highly influences the biodegradability properties of the hydrogels formed by crosslinking with gelatin [8]. In addition, gelatin-based films require plasticizers, which make the film stretch and soften. Glycerol is the most common plasticizer used, and its small hydrophilic molecule penetrates the gelatine protein chain, promoting the distances between the protein chains, and, consequently, direct interactions between protein chains turn weaker. That is why glycerol content directly affects mechanical properties [5]. As well, biodegradability is an essential parameter for an ideal food packaging material [9]; mechanical, thermal, and barrier properties are correlated with the packaging performance according to its protective functionality [10]. In addition, the manufacturing method of the film elaboration results in a variation in its physical and mechanical properties [11]. The casting method has broadly been used for film production due to its simplicity, in which simple steps are involved. Although it is not an industrial-scale method, it allows an approach to achieving the desired physical and mechanical properties of the films by shifting the concentrations of their main constituents [9]. Several studies are based on biodegradable gelatin–chitosan films [12,13], and their antimicrobial activity [14] has been reported. Furthermore, in recent years the focus of chitosan and gelatin films has been the addition of additives to improve their bioactive and barrier properties; without going into depth about the optimization of polymer concentrations to obtain an optimal film that can compete both in cost/efficiency and in mechanical properties with the packaging currently used by the food industry, such as low-density polyethylene (Table 1).

In addition, many types of biodegradable packaging have been investigated, focusing on the food industry, as a possible alternative to traditional packaging, which is based on low-density polyethylene. However, these proposed types of biodegradable packaging have mechanical and barrier properties that, in many cases, do not allow the replacement of LDPE for use in the food packaging industry. In this context, recent research by Hoque and Janaswamy propose packaging with a degradability rate greater than 90% in 30 days, although the high TS values and low EAB values do not allow its use as an alternative to polyethylene [23]. Also, research related to packaging based on biopolymers assumes that these are biodegradable, and associates this mainly with the origin of the components (Table 1), without this property being verified. On the other hand, and to the best of our knowledge, there is a lack of information allowing us to analyze the biodegradability of chitosan–gelatin based-films through an optimization of their components and considering how their properties are compromised, including the mechanical as well as barrier properties, considering LDPE as a reference material.

According to what was stated above, this research aims to verify the biodegradability of films made with CS/GEL/GLY, guaranteeing mechanical and barrier properties like LDPE, based on the optimization of their constituents. The film obtained under the optimal concentrations will be characterized in terms of its mechanical (TS and EAB) and barrier properties (swelling and solubility). The physicochemical properties were studied by Fourier-transform infrared spectroscopy, thermogravimetric analysis, scanning electron microscopy, and color. The biodegradability tests were carried out at the soil surface level, through a rapid methodology, as an approximation of a practical method that allows studying of the interactions of the film components over time, highlighting potential application in the food industry, as an alternative to non-biodegradable materials such as LDPE.

## 2. Materials and Methods

### 2.1. Materials

Food-grade chitosan (CS) of crustacean origin was used with a degree of deacetylation of ≥95% and a molecular weight between 150,000 and 300,000 g/mol (Quitoquímica, Concepción, Chile). The gelatin (GEL) used was from the skin of cold-water fish (Sigma Aldrich, Santiago, Chile), while the glycerol (GLY) had a 1.25 g/mL density (Sigma Aldrich, Santiago, Chile). The acetic acid used for the CS dilution had a molar mass of 60.05 g/mol (Emsure, Merck, Darmstadt, Germany). A low-density polyethylene bag (100 mm × 140 mm), approved for direct contact with food, was used as a control.

### 2.2. Experimental Design

A Response Surface Methodology (RSM) analysis was performed to optimize the content of CS, GEL, and GLY. A Box–Behnken design (BBD) was built with 15 runs, including three central points; the concentrations of CS (0.5, 1.0, and 1.5% *w*/*v*), GEL (0.5, 1.0, and 1.5% *w*/*v*) and GLY (0.25, 0.5 and 0.75% *w*/*v*) were selected as independent variables. The response variables were thickness, tensile strength (TS), and elongation at break (EAB). A polynomial model was fitted to the experimental data, and coefficients of fit (R^2^, adjusted R^2^, and predicted R^2^) were obtained. The experimental parameters were optimized following the desirability criteria (minimum thickness, maximum TS, and EAB) analyzed by Design-Expert statistical software (Version 11.0, StatEase, Inc., Minneapolis, MN, USA). The model was validated by preparing the sample under optimal conditions, which were subsequently characterized.

### 2.3. Film-Forming Solution (FFS)

The FFSs were formulated by casting method according to Liu et al., with some modifications [24]. Briefly, an amount of chitosan, according to the experimental design, was dissolved in acetic acid solution (1% *v*/*v*). The CS solution was stirred for 2 h at 3000 rpm until complete homogenization. After that, the corresponding amount of GEL and GLY was incorporated and further homogenized. The final FFS had a pH = 4.0 ± 0.1; this was measured using the following equipment: Adwa 1030, L Tech, Chile. Each FFS was prepared separately for each trial. Then, 15 g of FFS was cast on a square acrylic plate 10 cm wide and long, 3 mm thick, and with a 1 cm margin. Each film was recovered after solvent evaporation in an oven (Memmert UM400, Büchenbach, Germany) for 2 h at 60 ± 1 °C. The obtained films were stored in Petri dishes at room temperature (25 ± 1 °C) and 45% relative humidity (RH) until their characterization.

### 2.4. Attenuated Total Reflection—Fourier-Transform Infrared Spectroscopy (ATR-FTIR)

Fourier-transform infrared (FTIR) spectroscopy was recorded from wavenumber 4000 to 400 cm^−1^, using a PerkinElmer Spectrum Two (Bruker Banner Lane, Coventry, Germany) with an attenuated total reflectance (ATR) accessory. The analyses were recorded at room temperature (25 ± 2 °C) with 16 scans and a resolution of 4 cm^−1^.

### 2.5. Color of Film

The color of the films was measured according to the methodology proposed by Prus-Walendziak and Kozlowska [25] using a colorimeter (BCM 200, BIOBASE, Shandong, China) at room temperature with D65 illuminant. Before the measurements, the colorimeter was calibrated with a white standard (L* = 96.19, a* = +0.06, b* = −1.97). Results were expressed as L* (sample lightness), a* (−green to +red), and b* (−blue to +yellow) parameters. The total color difference (ΔE) and the whiteness index (WI) of the films were calculated using the following equations [26]:

∆E = [(∆L*)^2^ + (∆a*)^2^ + (∆b*)^2^]^0.5^ (1)

WI = 100 − [(100 − L*)^2^ + (a*)^2^ + (b*)^2^]^0.5^ (2)

where ΔL*, Δa*, and Δb* are the differences between the corresponding color parameters of the samples and that of a standard white plate used as the film background. Five readings were taken for each film at different points, and the average values were determined from the top and bottom sides.

### 2.6. Microstructure of Film

The film’s cross-sectional scanning electron microscopy (SEM) was obtained using a scanning electron microscope (JEOL, JSM-7401F, Tokyo, Japan). The film samples were measured at an acceleration voltage of 10 kV with a resolution of 1.0 nm. The films were freeze-breaking, using nitrogen, before the SEM examination.

### 2.7. Film Thickness

The thickness of the films (mm) was measured using a digital micrometer (E5010109, VETO & Co., Santiago, Chile). Five random locations around each film sample were used for the thickness determination.

### 2.8. Mechanical Properties

The mechanical tests of the films for the determination of the tensile strength (TS) and the elongation at break (EAB) were carried out using an Instron 3342 universal testing machine (Instron, Norwood, MA, USA) with a 500 N load cell (precision of 0.025 N). The films were approximately cut into 5 mm × 20 mm strips and stretched with a constant displacement speed of 0.5 mm/min. The tensile strain (also referred to as elongation) is computed as ε = *l*/*L*, where *l* is the instantaneous length, and *L* is the initial length (20 mm). The elongation can also be expressed as a percentage %ε = ε × 100. An approximation of real stress, due the absence of Poisson’s ratio data, is obtained assuming incompressibility of the material, and it is calculated as σ = *F*/*A* × (1 + ε), where *F* is the instantaneous force and *A* is the initial cross-sectional area of the samples. Measurements were performed in quintuplicate, and the average of each value was reported.

### 2.9. Swelling

The swelling of the film was assessed following the procedure outlined by Tagrida et al. [27]. The film was cut into pieces of 2 cm × 2 cm and then dried in an oven (Memmert UM400, Büchenbach, Germany) at 105 ± 1 °C for 24 h. Next, the dried film was weighed and submerged in 50 mL of distilled water for 1 h. After that, the excess water was removed by placing the film on a filter paper for 5 min and re-weighing it. Finally, the swelling percentage was calculated using the following formula:

Swelling (%) = (W_2_ − W_1_/W_1_) × 100 (3)

where W_1_ and W_2_ are the weight of the dried film and the weight of the film after eliminating the excess water, respectively.

### 2.10. Water Solubility

The solubility of the films was carried out at 25 °C according to the methodology described by Liu et al. [24]. Before the test, the films were dried as described in Section 2.9. The dried films were weighed and added to 50 mL of distilled water with continuous stirring for 24 h. Then, the film samples were filtered through filter paper (size 150 mm) and dried in an oven at 105 °C for 24 h. After this, the samples were weighed. The water solubility of the films was calculated according to the following equation:

Water solubility (%) = (W_i_ − W_r_/Wi) × 100 (4)

where W_i_ refers to the initial weight of the dry film and W_r_ is the weight of the dry film after immersion in water.

### 2.11. Thermogravimetric Analysis (TGA)

The Mettler Toledo TGA/DSC Model 2 StaRe System, fitted with a microbalance with ±0.1 μg precision, was used to examine the thermal stability, degradation, and alterations in the chemical composition of the interpenetrating network. Samples varying in weight from 4 to 8 mg were heated from 25 to 600 °C in a 70 μL alumina crucible with a scan rate of 10 °C min^−1^ under standard temperature and pressure conditions, controlling the nitrogen atmosphere and with a 40 mL min^−1^ flow rate.

### 2.12. Biodegradability Test

The biodegradability testing of the films was carried out according to the methodology proposed by Trejo-Caballero et al. [28]. Mentha piperita was used as an environmental model because it is a plant commonly found in Mexican gardens (where the trial was conducted) and because plastic waste is often inappropriately disposed of in its environment. The films were cut into dimensions of 1 cm^2^ and placed on the surface of the soil of the Mentha piperita pots for 31 days, with a 27–31 °C temperature and ca. 50–65% humidity. The physical and chemical changes of the films were determined for 31 days. For this, daily photographic monitoring of the samples was carried out, and the chemical composition of the samples was determined both at the beginning and end of the test using the FTIR methodology. LDPE was used as a control sample. Additionally, the weight loss percentage after 31 days of exposure was calculated. The tests were carried out in duplicate.

### 2.13. Statistical Analysis

Data were presented as mean ± standard deviation (SD). Analysis of variance (ANOVA) tests and the Tukey method considered a significance level set at *p* < 0.05, using Statgraphics CENTURION XVI. I (Manugistics Inc., Statistical Graphics Corporation, Rockville, MD, USA). All measurements were performed in triplicate.

## 3. Results and Discussion

### 3.1. Film Production: Optimization of CS, GEL, and GLY Content

**Figure 1:**
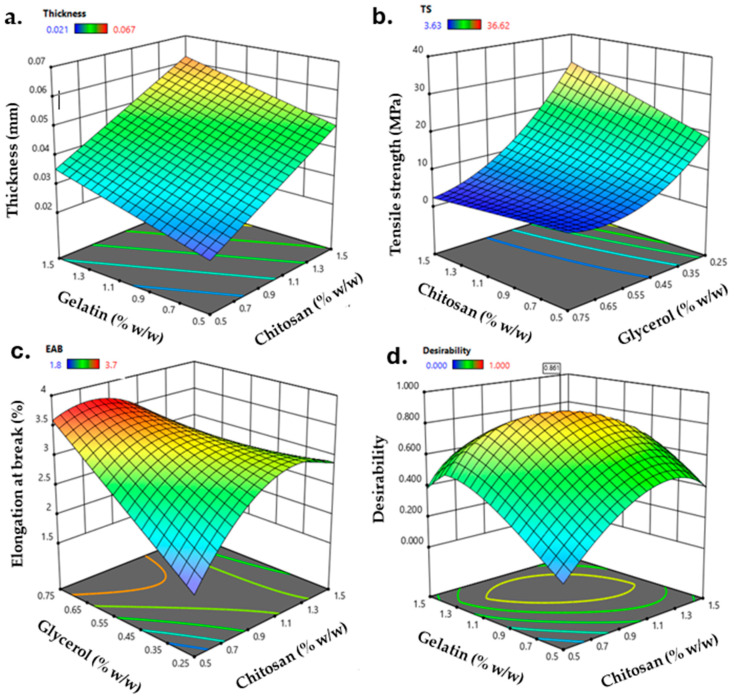
Response surface plots for (**a**) thickness (mm), (**b**) tensile strength (MPa), (**c**) elongation at break (%), and (**d**) contour curve for desirability as a function of the content of CS, GEL, and GLY.

The film production was carried out by casting, and a BBD was used to optimize the CS, GEL, and GLY content in the final film formulation. Mechanical properties are relevant parameters to preserve its integrity and reflect its tolerance of external stress during handling or transportation [3]. The thickness, TS, and EAB were selected as critical parameters associated with the film’s mechanical properties for the dependent variables. Figure 1 shows the surface response of the thickness (mm, Figure 1a), TS (MPa, Figure 1b), and EAB (%, Figure 1c) of the obtained films. The thickness fits a linear model in which the three independent variables are statistically significant (*p* < 0.05). The thickness shifted from 0.021 to 0.067 mm and, opposite to what was reported by Jridi et al. [29], the increment in CS content primarily increased the thickness of the film due to the interactions between GEL and CS molecules, resulting in a protruded network [27]. Also, the thickness was affected by the content of GLY, in which increasing the content of glycerol increases the thickness of the film because molecules of glycerol act as a filler in the resulting matrix, interacting with the film-forming polymers [30]. The TS (Figure 1b) ranged from 3.63 to 36.62 MPa, where the three independent variables have statistical significance (*p* < 0.05) and were fitted to a quadratic model. A higher TS contributes to the enhanced mechanical integrity of the packaging, and the TS obtained agrees with [30], confirming that the content of GEL and GLY highly influences TS. Based on this, mixing CS and GEL improves mechanical properties. While GEL promotes a more flexible film, the interaction with the semi-crystalline structure of CS promotes the formation of a more rigid structure. Therefore, incorporating GLY reduces the interactions between the polymer chains, resulting in a more flexible film [27]. The EAB of the obtained films ranged between 1.8% and 3.7%. EAB is highly influenced by the content of GLY (*p* < 0.05). The addition of GLY increases the capability of a film to stretch. However, a decrease in the EAB was observed for the higher content of GLY. This trend was argued for by Siripatrawan and Vitchayakitti, suggesting that a decrease in the EAB should be attributable to the crystalline structure formed by an excess of GLY in the CS structure, leading to a reduction in the flexibility of the film [31]. In addition, they refer to possible interactions between CS and a higher GLY content, leading to a crosslinking effect and decreasing the free volume and mobility of the chitosan chain, promoting a decrease in the EAB.

The optimization and validation of the model were conducted through numerical optimization for the desirability function. The overall desirability function (Figure 1d) was used to analyze the optimal conditions that led to the optimized film formulation (OPT-F). In this order, the maximum global desirability function was reached (0.86) when CS, GEL, and GLY content were 1.1%, 1.1%, and 0.4% (*w*/*v*), respectively. Under these conditions, the predicted variable values are shown in Table 2.

The predicted and experimental data of the optimized conditions were compared as an essential step to validate the model. The differences between the predicted and the experimental values ranged from 6.98% for thickness to 18.75% for EAB, guaranteeing successful responses in the region where they were optimized.

### 3.2. Chemical Composition of the Films

**Figure 2:**
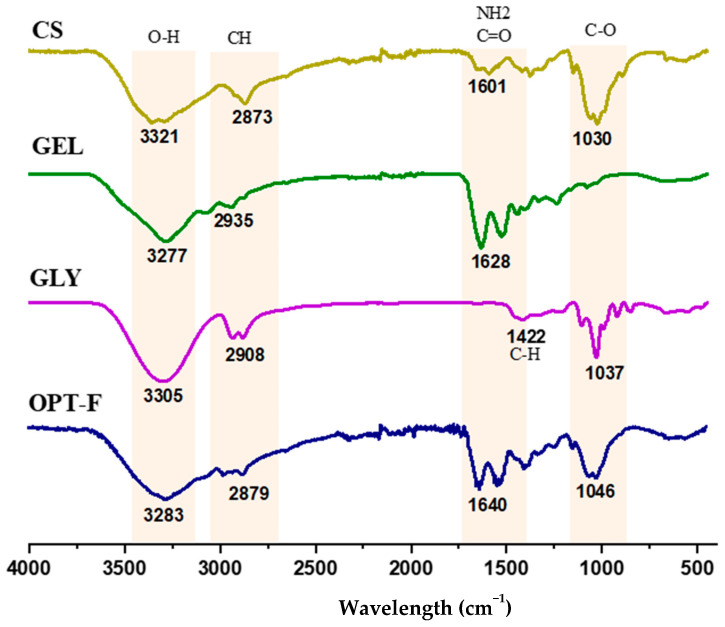
FTIR spectra of chitosan-based film (OPT-F) and comparison with its single constituents.

Fourier-transform infrared spectroscopy (ATR-FTIR) analyses were carried out to evaluate possible structural changes and physical or chemical interactions by CS, GEL, and GLY functional groups for a deeper understanding of the interactions of the CS and GEL polymer chains and GLY and their role in the modification of the mechanical properties of the film. Hence, the relevant transmittance peaks at wavenumbers from 4000 cm^−1^ to 400 cm^−1^ for the optimized film (OPT-F) were compared with individual components to examine shifting and interaction among its constituents (Figure 2). The position of the most relevant peaks in the spectra agrees with previous studies reported by other authors [18,32,33]. The bands in the wavelength range between 1030 and 1046 cm^−1^ in the CS and GLY, and the OPT-F samples correspond to the compounds’ fingerprint, referring to the C-O stretching groups [32,34]. In the spectrum of GLY, a peak is observed at 1422 cm^−1^ related to the stretching of CH, specifically with the movement of CH_2_ from proline and glycine [32]. Additionally, the OPT-F and the compounds CS, GEL, and GLY displayed a large peak in the 3305–3321 cm^−1^ range that was attributed to O–H stretching vibrations [18,32]. Likewise, in the range of 2873 to 2935 cm^−1^, the presence of the Amida B group is observed where the CH groups of antisymmetric and symmetric stretching are found [32,34]. Finally, in the region located at 1601–1640 cm^−1^, the interaction between the CS and GEL is observed. Relevant peaks in the region 1500–1700 cm^−1^ were associated with electrostatic interaction between the amino and carbonyl groups [33]. The above is confirmed since in the OPT-F, it is observed that the peak increases to 1640 cm^−1^ concerning the CS and GEL, which are 1601 and 1628 cm^−1^, respectively, indicating the formation of electrostatic interactions between the polymers used to formulate the optimized film, forming a homogeneous layer.

### 3.3. Analysis of Film Color

Optical properties such as color and transparency are relevant for film used as packaging in the food industry due to it altering the appearance of the coated product [29]. Also, sensory properties and consumer acceptance are affected by the color of the packaging [32]. In the present study, the appearance of the optimized film was compared with LDPE. For the OPT-F and LDPE (Table 3), the lightness has statistically significant differences (*p* < 0.05), with a clear tendency for a slight appearance in the optimized film. This trend agrees with reports by Jridi et al. for films containing GEL [29]. Relative to redness (a*), no statistical significance (*p* > 0.05) was observed. At the same time, OPT-F’s yellowness (b*) did present a statistical significance compared to the LDPE, indicating that OPT-F has a greater tendency to yellowness than LDPE. Similar behavior was reported by Chu et al. for bi-layer films of CS and GEL, where the yellowish tendency is associated with the presence of β-1-4 linked 2-amino-2-deoxy-d-glucopyranose repeated units [33]. The whiteness index (WI) measures the amount of light reflected by a surface through the visible light spectrum, which helps determine how white a surface appears to the human eye. It is expressed as a percentage on a scale of 1–100%, with 100% being the value that should correspond to a perfect white. The WI values of the OPT-F presented statistically significant differences with LDPE (as seen in Table 3), where OPT-F has a more reflective surface than LDPE, and this is associated with the interaction between CS and the other constituents [35].

### 3.4. Morphology and Microstructure of Designed Chitosan-Based OPT-F Film

**Figure 3:**
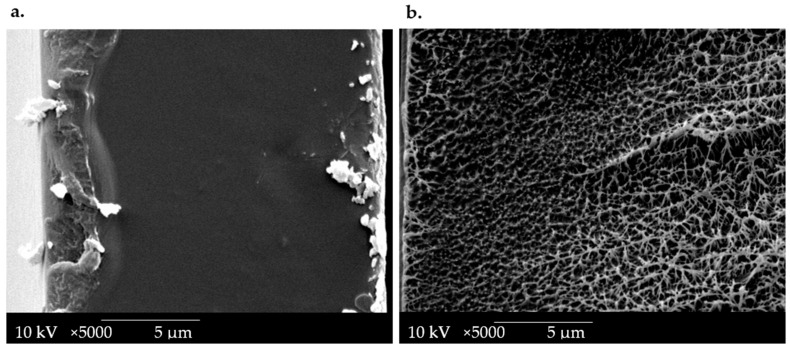
SEM image of the cross-section of (**a**) OPT-F and (**b**) LDPE.

Scanning electron microscopy (SEM) micrographs of cross-sections of the OPT-F film were performed to evaluate the designed material’s microstructure and compare it with LDPE film, as presented in Figure 3. It can be seen that the LDPE cross-sections at 5000× (Figure 3b) revealed a heterogeneous morphology, with porosity. This behavior reveals that significant morphological irregularities were more common in LDPE films with a greater thickness, according to the report by Szlachetka et al. [36]. The OPT-F observed at 5000× (Figure 3a) shows a homogeneous, non-porous structure with good structural integrity due to the high compatibility of each of the components. These attributes, mainly attributable to the hydrophilic nature of the main components, correlate with the good compatibility and compact morphology of the obtained films [18,33]. Even though the microstructures of LDPE and OPT-F cannot be compared due to their different production processes, a homogenous film is generated for possible use as leading food packaging by combining CS, GEL, and GLY.

### 3.5. Mechanical Properties

**Figure 4:**
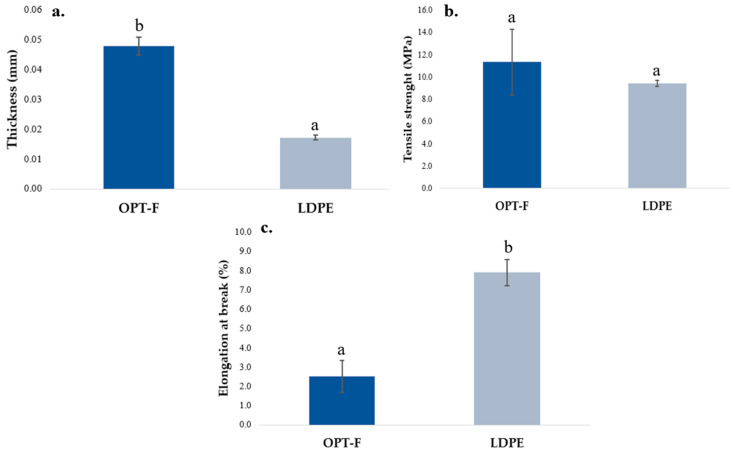
Comparison of the mechanical properties of chitosan-based OPT-F with LDPE: (**a**) thickness, (**b**) TS, and (**c**) EAB. Different letters indicate significant differences (*p* < 0.05).

The adequate performance of the mechanical properties of the films is relevant and desirable when they are used as food packaging [29]. The mechanical properties of OPT-F and the LDPE film as control are compared in Figure 4. A significant difference (*p* < 0.05) is observed for thickness, OPT-F being thicker than the LDPE film (Figure 4a). Jridi et al. obtained films by casting using GEL and CS with a thickness closer to 0.050 ± 0.003 mm and concluded that adding CS to the matrix reduces the thickness of the film [29]. However, the differences between the OPT-F and LDPE films are highly influenced by the film production methods in which extrusion, for LDPE, considerably reduces the thickness of the film [37]. For TS (Figure 4b), no significant differences were observed for both the OPT-F and LDPE films. In addition, the TS obtained for the biodegradable OPT-F agrees with reports by Luzi et al. for LDPE (8–31 MPa) [38]. This result supports the idea of using biodegradable materials for film production able to be used as food packaging, providing a similar mechanical integrity as LDPE films that guarantees the integrity of the content, avoiding food deterioration in transportation, handling, or storage operations [39]. Khan, Ezati, and Rhim obtained chitosan–gelatin-based film with a TS higher than 62 MPa for 2% (*w*/*w*) of chitosan, 2% (*w*/*w*) of gelatin and 30% (*w*/*w*) of glycerol [16]. A similar trend in TS was reported by Haghighi et al. [40], who formulated films based on chitosan–gelatin blends, and they reported a TS up to five times higher than that obtained in this research, which is considered by them as very high for application in the packaging industry. Consequently, the optimized concentrations of chitosan and gelatin for OPT-F guarantee properties similar to LDPE, and being higher than 3 MPa, they can be used in the packaging industry, ensuring the integrity of the film.

For OPT-F, the EAB was lower than LDPE (*p* < 0.05), as observed in Figure 4c. Films with a higher TS and lower EAB show the typical behavior of non-plasticized films [41]. The EAB results agree with those reported by Chang et al., and could be attributable to the lower content of GLY as a plasticizer [42]. Despite this, the OPT-F film could be applied as alternative natural-based food packaging, considering some limitations in its application according to the nature of the food matrix.

### 3.6. Swelling

The degree of swelling is a relevant parameter within the barrier properties of the films, because it determines the ability of the polymer matrix to maintain its structure in the presence of water [43]. Generally, a low percentage of swelling is desirable for films with a packaging application [44]. Table 4 shows that the degree of swelling in OPT-F and LDPE are statistically different. This is due to the chemical nature of OPT-F obtaining a higher swelling degree compared to LDPE, these being 75.95% and 2.68%, respectively. The high degree of swelling in OPT-F is caused by the hydrophilic nature of gelatin and glycerol, since they react with water molecules through hydrogen bonds [45]. In the study carried out by Zhang, Han, and Zhou, they found that the swelling degree of chitosan and gelatin films was 6611.4% and they reduced it to 314.1% by adding 15% wt of citric acid [18]. Therefore, in this study we reduced the swelling degree by optimizing the concentrations of chitosan, gelatin, and glycerol, obtaining a film with greater stability to water contact. Furthermore, the formulation proposed in this study serves as a basis for the future addition of crosslinking agents that will allow the % of swelling to be further reduced, without compromising the degradability of the film.

### 3.7. Water Solubility (WS)

Table 4 shows the WS of the OPT-F and LDPE samples, obtaining values of 24.3% and 0.9% respectively. From this, it was determined that the WS of the analyzed samples are statistically different from each other. In the same way as discussed in Section 3.6. this is due to the hydrophilic nature of gelatin and glycerol. However, in this study, a lower percentage of WS was obtained compared to other studies of chitosan and gelatin films. For example, Reyes Mendez et al. reported a WS of 33.7% and added eugenol essential oil to improve the solubility, reaching a WS of 35.8% [17]. The chitosan and gelatin film obtained here, OPT-F, led with a WS of 39.8% [18]. Therefore, the optimization of the concentrations of chitosan, gelatin, and glycerol proposed in this study allowed the WS values to be reduced.

### 3.8. Thermogravimetric Analysis

**Figure 5:**
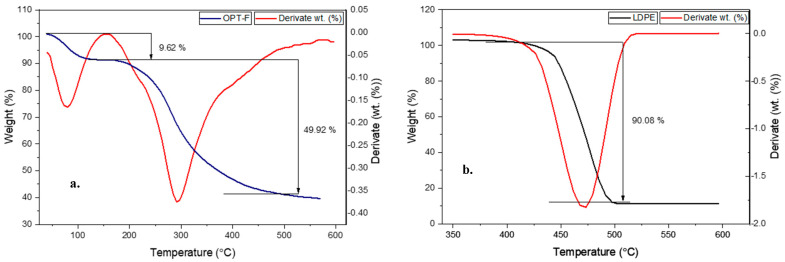
Thermal gravimetric analysis (TGA) and differential thermal analysis (DTA) curves for (**a**) OPT-F and (**b**) LDPE.

Thermogravimetric analysis evaluates a material’s thermal stability, including the sample’s moisture or volatile compound content. In this regard, a TGA measurement was performed to evaluate the thermal stability of the designed CS/GEL/GLY film and compare it with the thermal stability of LDPE, as shown in Figure 5. The OPT-F exhibits two main mass losses closer to 100 °C and 300 °C, respectively (Figure 5a). On the other hand, for the LDPE, mass losses ca. 90.08 wt. % were observed at 450 °C (Figure 5b), suggesting high thermal stability under environmental conditions, a key factor of its poor biodegradability. According to the TGA and DTA curves for LDPE films (Figure 5b) a single decomposition step closer to 450 °C is attributable to polymers in which C-C bonds prevail in the main chain, promoting a break in the polymeric chain and further thermal decomposition [46]. For CS/GEL films, the first mass losses were observed for temperatures lower than 150 °C and could be attributable to physisorbed water and traces of acetic acid [18], and they agree with the temperature obtained for the OPT-F film in this research. A similar trend was observed in both the CS and GEL thermograms (Supporting Information, Figure S1). The second relevant mass loss was closer to 300 °C in the OPT-F (49.9%) and was closer to the temperature in which CS (300 °C) and GEL (320 °C) had the most relevant mass losses. Since no intermediate peaks were observed at temperatures above 150 °C, it can be stated that the system behaves as a homogeneous mixture with a good miscibility of its single constituents [29], also confirmed by the FT-IR analysis, in which intermolecular hydrogen bonds formed by the interaction of CS, GEL, and GLY promoted the thermal stability of the film. As the OPT-F degradation temperature was higher than 300 °C, it is feasible to confirm that the obtained film can be subjected to packaging and thermal processing temperatures below 300 °C, which are characteristic in the food industry, without causing thermal decomposition of the film [47], as occurs with materials such as LDPE. 

### 3.9. Biodegradability Test of Designed OPT-F

**Figure 6:**
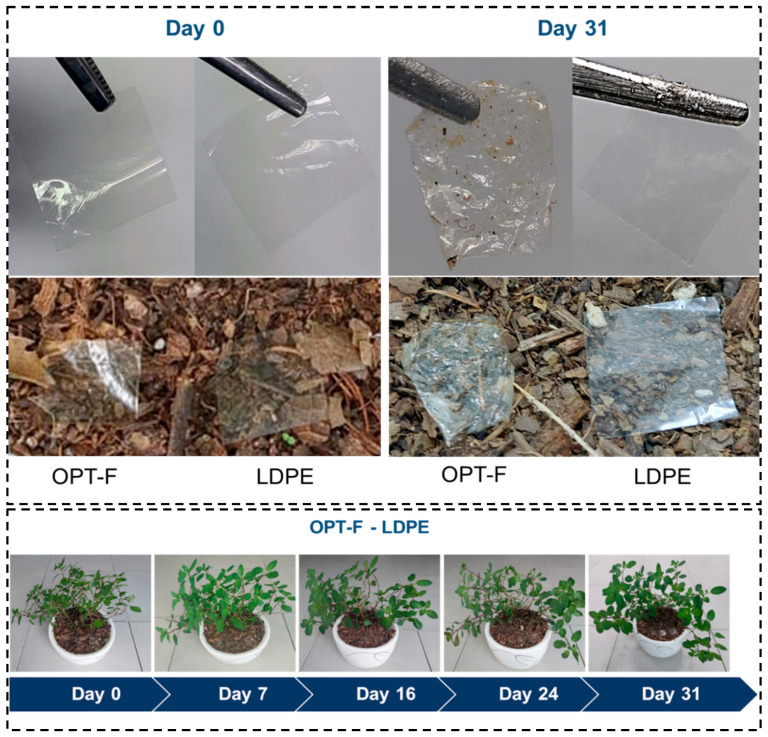
Biodegradability tests for both OPT-F and LDPE films when exposed to the soil surface of a Mentha piperita pot as an environmental model.

The OPT-F and LDPE films were exposed to the soil surface of *Mentha piperita* pots for 31 days (Figure 6) to study the change in the samples’ color, mass, dimensions, and composition. After 31 days, significant physical changes were observed for the OPT-F film, since the sample wrinkled and small holes formed, indicating the beginning of degradation [48]. In contrast, no morphological changes were observed for the LDPE film when it was compared for day 0 and day 31. Regarding the color of the samples, OPT-F presented changes in color from day 0 to 31, becoming more yellowish, while LDPE did not present color changes. A similar situation occurred with the mass loss percentage, where the OPT-F had 7.08%, while LDPE did not present a mass loss. Therefore, the LDPE film did not show signs of degradation (Table 5). Also, it is important to mention that the mass values for the OPT-F sample include the pot soil’s residues adhered to the sample’s surface, which affected the weight measurements, and possibly the mass loss on day 31 was higher. In a study carried out by Oberlintner et al. on CS films, a mass loss of more than 80% was obtained in 7 days [49], while in a study carried out by Martucci and Ruseckaite on GEL films, a mass loss of 18% was obtained in 3 days [50].

This study obtained a lower degradation percentage than those reported in the literature, possibly due to the homogeneous structure that is formed from the mixture of CS and GEL. Furthermore, many factors such as soil type and microbial community also influenced the degradation of the proposed film [51]. However, the OPT-F sample shows a more significant mass loss compared to LDPE.

**Figure 7:**
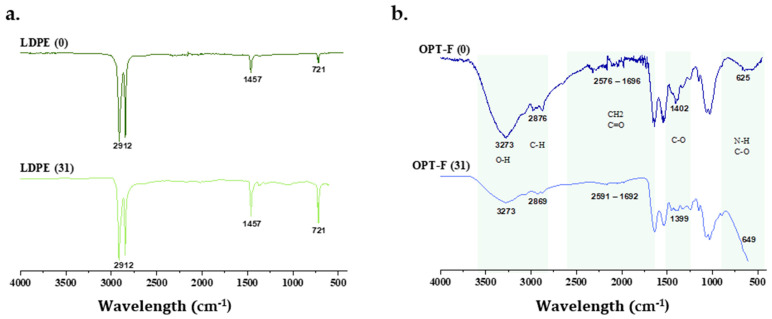
FTIR spectra of (**a**) chitosan-based OPT-F and comparison with (**b**) LDPE after 31 days of exposure to the soil surface of Mentha piperita pots.

There are studies that analyze the percentage of degradation of films that are based on biopolymers, by calculating the mass loss that the film suffers when exposed to environmental conditions [23,52]. While this study also analyzed the changes in the structure of OPT-F and LDPE films after 31 days of exposure to the soil surface by FTIR spectroscopy. In Figure 7, it is observed that the structure of LDPE (Figure 7b) on day 31 had no structural changes compared to day 0, indicating that this material, when exposed to the soil surface, maintains its chemical structure, indicating that its polymer chain does not break and, consequently, there is no degradation on the soil surface. However, OPT-F did present changes in its chemical structure on day 31 of being exposed to the soil surface (Figure 7a), demonstrating that after 31 days of exposure, a decrease in the O–H groups (3273 cm^−1^) and the C-H groups (2869 cm^−1^) is presented, suggesting the biodegradation of the designed film. Also, significant changes in CH_2_, C=O, NH, and C-O bonds were observed [53]. The above indicates that polymer chain breakdown through chemical hydrolysis forms microdoses of O_2_, CH_4_, water, biomass, humic matter, and other natural substances [54]. As observed in Figure 6, it could be inferred that with the degradation of OPT-F, no qualitative changes are observed for the Mentha piperita, suggesting that the growth integrity of the plant is not compromised.

## 4. Conclusions

An eco-friendly and biodegradable chitosan-based film (OPT-F) was obtained by optimizing the content of CS, GEL, and GLY using a Response Surface Methodology, with a similar mechanical performance compared with non-degradable LDPE. As a result, for the optimized CS (1.1% *w*/*v*), GEL (1.1% *w*/*v*), and GLY (0.4% *w*/*v*), the thickness, TS, and EAB obtained were 0.046 mm, 11.48 MPa, and 2.6%, respectively. Under these conditions, the OPT-F was thermally stable and desirable for packaging submitted to a high thermal process. The biodegradability test revealed that OPT-F, after 31 days of exposure in the soil surface, suffered chemical changes in the CH2, C=O, NH, and C-O bonds, suggesting the biodegradation of the film, while LDPE did not present any chemical changes in its structure. The optical properties suggested the optimized film’s homogeneity, enhancing the protected food’s visual properties. This research demonstrates the potential applicability of biopolymer-based films as a novel alternative in the food packaging industry, avoiding pollution and offering mechanical, thermal, and biodegradability properties that promote food preservation, handling, and storage. However, a long-term study is required to determine the complete degradation time of the films. With this starting point, an optimized formulation could include crosslinking or the incorporation of bioactive compounds, to improve the activity, mechanical properties, and swelling of the film without compromising its degradability.

## Figures and Tables

**Table 1:** Chitosan–gelatin films in food packaging.

Film-Forming Components	Application	Control Film	Principal Results	Ref.
Chitosan–gelatin–thymol–deep eutectic solvent	Food packaging	Chitosan–gelatin film	The solvent improved the barrier properties of the resulting films, as well as their mechanical and bioactive properties.	[15]
Chitosan–gelatin–green tea carbon dots	Food packaging	Chitosan–gelatin film	Quantum dots presented good compatibility with the polymer matrix, resulting in homogeneous films with bioactive properties. However, the quantum dot concentration affects the elongation percentage.	[16]
Chitosan–gelatin-eugenol and/or oregano essential oil	Food packaging	Chitosan–gelatin film	The addition of essential oils confers antimicrobial properties on *S. aureus* and *E. coli*. No mechanical properties were analyzed.	[17]
Chitosan–gelatin–citric acid–red cabbage pigment–enterocin	Food packaging	Chitosan–gelatin film	A smart active compound with antioxidant properties, pH responsiveness, and bacterial inhibition was obtained.	[18]
Chitosan–gelatin–glycerol films		Chitosan–gelatin film	Mixing chitosan and gelatin at a 1:1 ratio resulted in materials with better mechanical and barrier properties.	[19]
Chitosan–gelatin–*Ferulago angulate* essential oil	Food packaging	Chitosan–gelatin film	Essential oil confers antimicrobial properties and improves water vapor permeability. However, the tensile strength decreases when essential oil is incorporated into the polymer matrix.	[20]
Chitosan–gelatin–grape seed extract and/or jabuticaba peel	Food packaging	Chitosan–gelatin film	Grape seed and jabuticaba peel extracts improved the antioxidant properties of the films; however, the mechanical properties were not characterized.	[21]
Chitosan–gelatin–apple peel nanoparticles	Food packaging	Chitosan–gelatin film	Apple peel nanoparticles improve the antioxidant and physical properties of the film. However, its barrier properties decrease.	[22]

**Table 2:** Predicted and experimental response values of the designed CS/GEL/GLY film.

Response	Predicted Value	Experimental Value	Absolute Residual Error (%)
Thickness (mm)	0.043 ± 0.003	0.046 ± 0.003	6.98
Tensile strength (mPa)	13.00 ± 1.15	11.48 ± 1.42	11.73
Elongation at break (%)	3.2 ± 0.2	2.6 ± 0.3	18.75

**Table 3:** Color parameters (L*, a*, and b*), total color difference (ΔE*), and whiteness index of the OPT-F film and their comparison with LDPE.

Sample	Color Parameters				
	L	a	b	ΔE	WI
OPT-F	95.68 ± 0.28 ^a^	−0.21 ± 0.21 ^a^	1.63 ± 0.31 ^a^	3.65 ± 1.05 ^a^	95.37 ± 0.37 ^b^
LDPE	91.01 ± 0.47 ^b^	−0.17 ± 0.27 ^a^	−1.61 ± 0.22 ^b^	5.20 ± 0.47 ^b^	90.85 ± 0.44 ^a^

The values are presented as means ± SD. Different letters in the same column indicate significant differences (*p* < 0.05).

**Table 4:** Swelling and solubility of OPT-F film and comparison with LDPE.

Sample	Swelling (%)	Water Solubility (%)
OPT-F	75.95 ± 5.03 ^b^	24.34 ± 2.47 ^b^
LDPE	2.68 ± 1.31 ^a^	1.8 ± 0.7 ^a^

The values are presented as means ± SD. Different letters in the same column indicate significant differences (*p* < 0.05).

**Table 5:** OPT-F and LDPE weight loss in the soil of a Mentha piperita pot.

Sample	Initial Weight (mg)	Final Weight (mg)	Weight Loss (%)
OPT-F	11.3 ± 0.3	10.5 ± 0.6	7.1
LDPE	11.4 ± 0.4	11.4 ± 0.4	0

## Data Availability

Data are contained within the article.

## Supplementary Materials

The following supporting information can be downloaded at: https://www.mdpi.com/article/10.3390/polym16172471/s1, Figure S1. TGA measurements of CS, GEL, and GLY components to design of chitosan-based OPT-F.
