# Supplementary material for: Eco-Friendly Design of Chitosan-Based Films with Biodegradable Properties as an Alternative to Low-Density Polyethylene Packaging
Source: Polymers (Basel). 2024 Aug 30;16(17):2471. doi: 10.3390/polym16172471 (PMC11398076; doi:10.3390/polym16172471)

**Supporting information**

**Figure S1.** TGA measurements of CS, GEL, and GLY components to the design of chitosan-based OPT-F


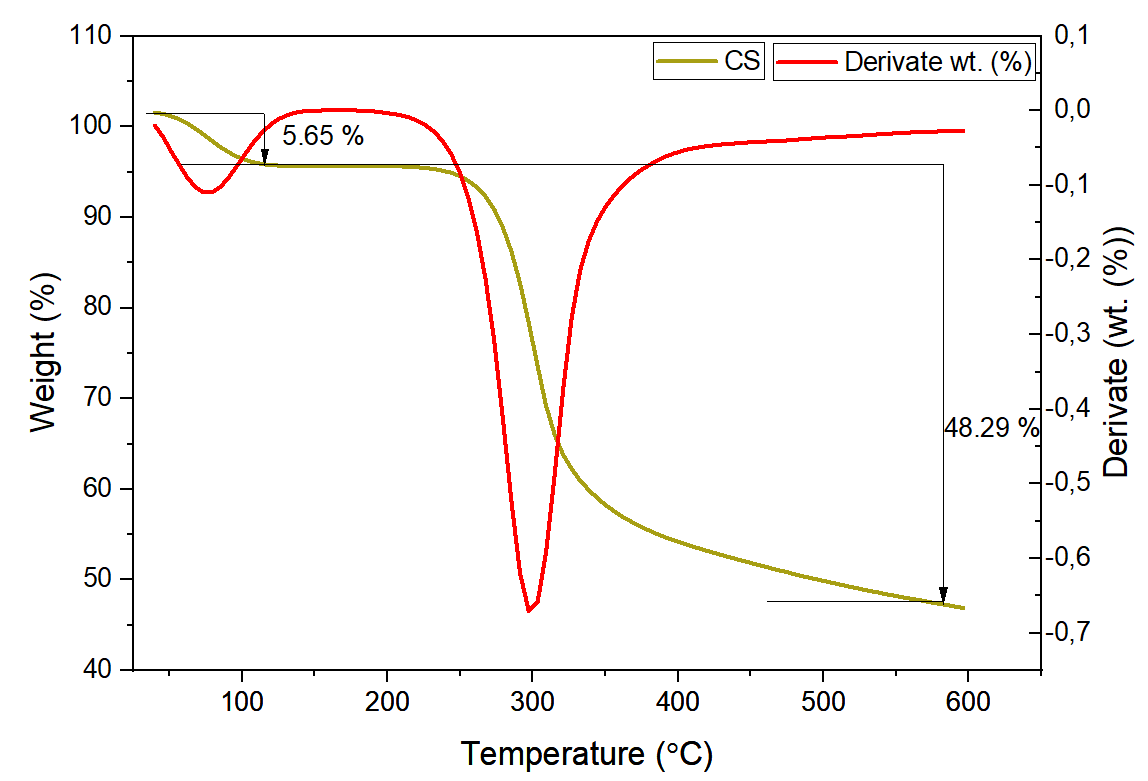


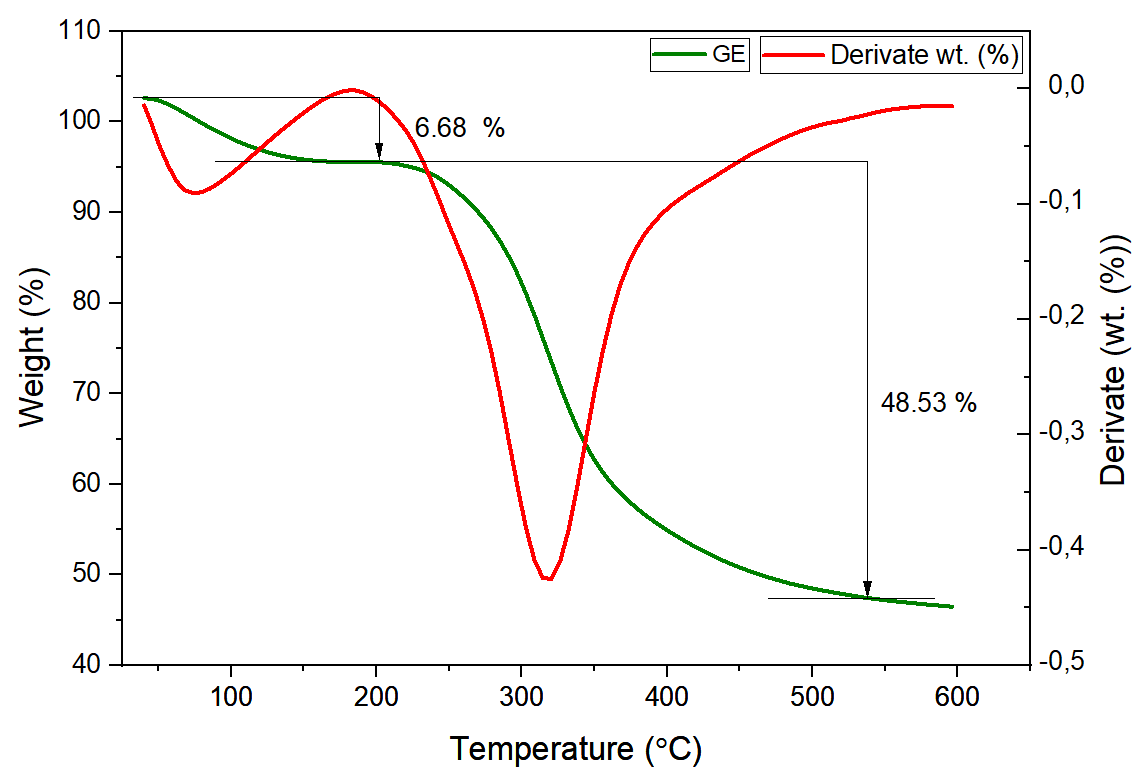


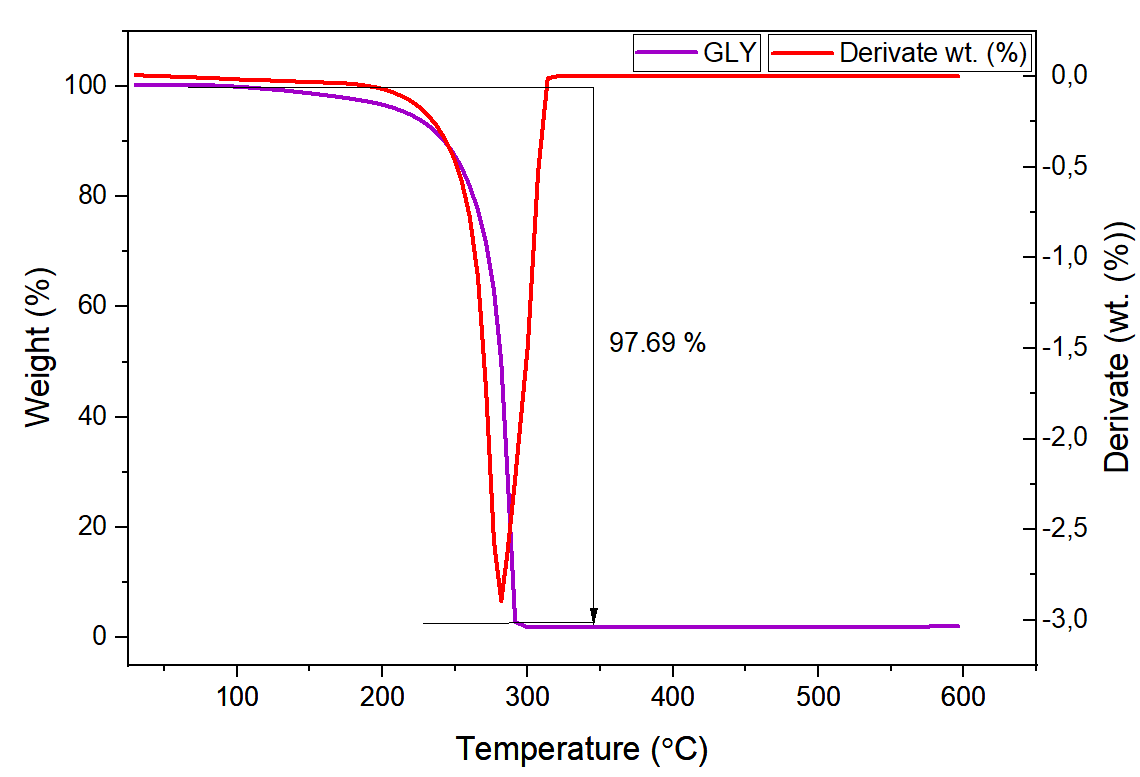

Supplement: Supplementary file 1 [file polymers-16-02471-s001.zip › polymers-3174643-supplementary.docx]
